# *In utero* Head Circumference is Associated with Childhood Allergy

**DOI:** 10.3389/fped.2015.00073

**Published:** 2015-09-08

**Authors:** David P. Eviston, Anna Minasyan, Kristy P. Mann, Dianne E. Campbell, Ralph K. Nanan

**Affiliations:** ^1^Discipline of Obstetrics, Gynaecology and Neonatology, Charles Perkins Centre Nepean, Sydney Medical School Nepean, The University of Sydney, Penrith, NSW, Australia; ^2^Discipline of Paediatrics and Child Health, Charles Perkins Centre Nepean, Sydney Medical School Nepean, The University of Sydney, Penrith, NSW, Australia; ^3^NHMRC Clinical Trials Centre, The University of Sydney, Camperdown, NSW, Australia; ^4^Department of Allergy and Immunology, The Children’s Hospital at Westmead, The University of Sydney, Westmeabd, NSW, Australia; ^5^Discipline of Paediatrics and Child Health, The University of Sydney, Penrith, NSW, Australia

**Keywords:** allergy, developmental programming, fetus, head circumference, mid-gestation, ultrasound

## Abstract

**Background:**

Altered fetal growth is known to be associated with allergic disease. Specifically, increased head circumference at birth has been linked to asthma and elevated IgE. However, few studies have examined a link between early fetal anthropometry and allergic disease. The aim of this study was to examine head circumference at mid-gestation in children diagnosed with allergy.

**Methods:**

This was a retrospective cohort study, comprising pregnancies delivered between 10/2006 and 9/2010 at Nepean Hospital, Australia. Exclusion criteria were illegal drug use, alcohol consumption, gestation <35 weeks, and gestational hypertension. Pregnancy data were sourced from the Nepean Obstetric Database. Atopic diseases (asthma, atopic dermatitis, and IgE-mediated food allergy) were assessed by questionnaire at age 1–5 years. Infants from pregnancies with completed questionnaires, who also had a mid-gestation ultrasound scan, were included (*N* = 121). Multiple logistic regression techniques were used to model head circumference against the development of allergies.

**Results:**

Smaller head circumference at mid-gestation was associated with increased odds of allergic disease in children aged 1–5 years. A 1 mm smaller head circumference was associated with a 7% increased chance of allergies being later diagnosed, adjusted for gestation (95% CI: 1–14%, *p* = 0.036). Head circumference at mid-gestation was also inversely correlated with the presence of multiple atopic disease.

**Conclusion:**

Smaller mid-gestational head circumference is associated with early childhood allergic disease, which suggests that fetal programing of allergic disease occurs before mid-gestation. This suggests that mediators such as brain-derived neurotrophic factor may be dysregulated early *in utero* in a milieu, which also predisposes to atopic disease.

## Introduction

Allergic disease is likely programed *in utero*, as a product of altered fetal growth and development (1). In support of this theory, changes in fetal anthropometry, including decreased birth weight (2) and increased head circumference at birth (3), have been previously reported to be associated with asthma. Furthermore, an increased head circumference at birth has been linked to elevated IgE levels in later life (1, 4). Associations between fetal growth disturbance and allergy have previously been attributed to “brain sparing,” whereby head growth is maintained in the nutrient-starved fetus at the expense of less critical organs, including the thymus (5). Accordingly, impaired thymus development may have long-term immune consequences, including an increased risk of allergy. However, in asthma, head and thymus size are positively correlated and thymus size appears unrelated to asthma (6), suggesting an alternative mechanism.

More than adequate nutrient supply, brain growth depends on neurotrophins, including brain-derived neurotrophic factor (BDNF) (7). In addition to their functions in neurodevelopment, neurotrophins are present in immune cells and are highly active in allergic disease (8, 9). Furthermore, BDNF levels correlate with disease severity in asthma (10), atopic dermatitis (11, 12), and allergic rhinitis (13). Differential exposure to neurotrophins *in utero* may therefore explain altered fetal growth in allergic disease.

Fetal anthropometry at mid-gestation has not been well studied in allergy. Hence, the aim of this study was to investigate fetal head circumference at mid-gestation in allergic disease. It was hypothesized that fetal head size would be altered in fetuses who were subsequently diagnosed with allergic disease in early childhood.

## Materials and Methods

This was a retrospective cohort study, which included subjects from a larger pre-eclampsia cohort study (Eviston et al; unpublished). Singleton pregnancies delivered between 1/10/2006 and 30/9/2010 at Nepean Hospital, Australia were considered for inclusion. Exclusion criteria included gestational hypertension, illegal drug use, alcohol consumption greater than one standard drink per day, gestation at delivery <35 weeks, and birth weight <2000 g. Eligible pre-eclamptic pregnancies were matched with non-pre-eclamptic pregnancies (1:2), according to maternal age, gestation, smoking status, and child’s date of birth.

All procedures contributing to this work comply with the ethical standards of the relevant national guidelines on human experimentation [National Statement on Ethical Conduct in Human Research (2007)] and with the Helsinki Declaration of 1975, as revised in 2008, and approval was granted by The Ethics Committee of the Sydney West Area Health Service.

Current and past allergic disease was assessed via Comprehensive Early Childhood Allergy Questionnaire (CECAQ), as below (Appendix A), or a structured phone interview in the event of no reply. CECAQ is a combined questionnaire designed to detect three common atopic conditions in the age group of 1–5 years (asthma, atopic dermatitis, and IgE-mediated food allergies) and is based on other validated questionnaires, namely the Food Allergy Questionnaire (14), the ISAAC questionnaire plus its validated modification (15, 16), and the UK Working Party criteria for diagnosing atopic eczema (17).

**Figure 1:**
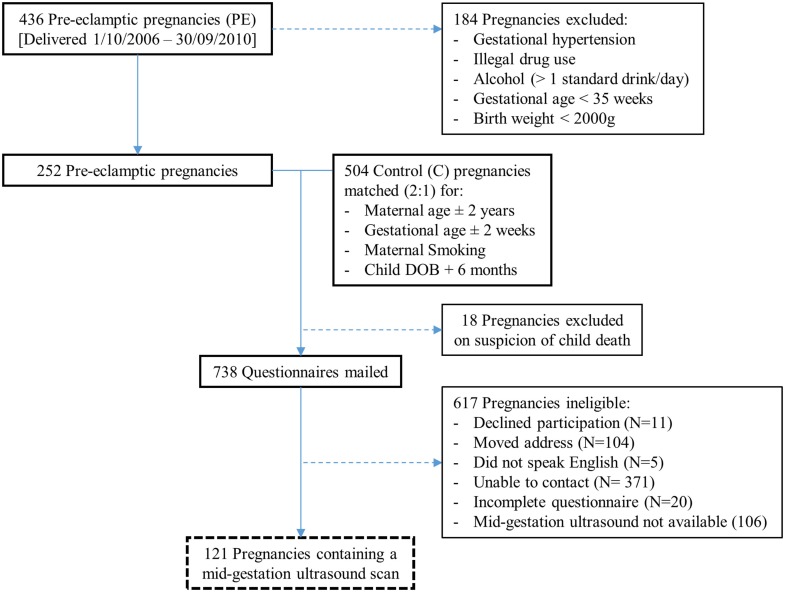
**Flow of pregnancies for inclusion**.

During the data collection period, 15,273 singleton pregnancies were delivered at Nepean Hospital, which included 436 pre-eclamptic pregnancies. Based on the exclusion criteria, 184 of these pregnancies were ineligible (Figure 1). The remaining 252 pre-eclamptic pregnancies were matched (1:2) with healthy control pregnancies (504). Eighteen pregnancies were excluded on suspicion of child death, and a total of 738 allergy questionnaires were mailed. Following an attempt at telephone correspondence for non-responders, 227 completed questionnaires were collected. Utilizing Viewpoint software, 121 of these pregnant mothers were found to have a mid-gestation ultrasound scan and were included in the final cohort.

Head circumference measurements were obtained by clinical sonographers, as part of a standard mid-gestation ultrasound protocol. Gestational age was based on an earlier dating scan, or the first day of the last menstrual period, if a dating scan was not available.

Pre-eclampsia was defined according to recommendations proposed by the International Society for the Study of Hypertension in Pregnancy (18).

Case matching pre-eclamptic pregnancies was performed to identify a possible link between pre-eclampsia and childhood allergy. Control pregnancies, for each pre-eclamptic case, were the first two pregnancies encountered from a random list of eligible pregnancies.

Continuous maternal and fetal characteristics were compared between atopic and non-atopic groups using two-tailed *t*-tests (or Mann–Whitney *U*-tests where the normality assumption was violated). Categorical characteristics were compared using either Fisher’s exact or Chi-square tests, as appropriate to group size. Similar techniques were used to compare infant variables between pre-eclamptic and non-pre-eclamptic pregnancies.

Logistic regression techniques were used to model the development of allergies (Y/N). Univariate models were initially performed, with variables significant at *p* < 0.20 considered for multivariate modeling (Table 2). A multivariate model was performed which included mid-gestational variables (head circumference at mid-gestation, gestation at ultrasound). The effect of allergy was also investigated with ordinal logistic regression techniques to model the development of no allergy vs. one allergy vs. two or more allergic diagnoses (asthma, atopic dermatitis, and IgE-mediated food allergy) at mid-gestation. The assumption of proportional odds was met. No adjustments have been made for multiple comparisons. A two-sided *p* value < 0.05 was considered statistically significant. All modeling was performed in SAS v9.3 (SAS Institute Inc., Cary, NC, USA).

## Results

Allergic disease and non-allergic disease groups contained a relatively large, but comparable, number of pre-eclamptic pregnancies (34.1 vs. 36.3%, *p* = 0.81; Table 1). Similar demographic variables were noted between infants born to pre-eclamptic (43) and non-pre-eclamptic pregnancies (78), including male baby (53.5 vs. 51.3%, *p* = 0.85), any allergic disease (32.6 vs. 34.6%, *p* = 0.84), asthma (16.3 vs. 21.8%, *p* = 0.63), atopic dermatitis (18.6 vs. 12.8%, *p* = 0.43), food allergy (9.3 vs. 9.0%, *p* = 1.0), and median child age (2.3 vs. 2.6 years, *p* = 0.42), respectively. Head circumference at mid-gestation was also comparable (pre-eclamptic = 163.5 mm, non-pre-eclamptic = 163.2 mm, *p* = 0.86). Atopic disease was unrelated to pre-eclampsia in univariate analysis (Table 2).

**Table 1:** **Participant characteristics**.

Characteristic	Allergy (*n* ***=*** 41)	No Allergy (*n* ***=*** 80)	*P* value
**Mother**
Age (yr)	28.9 (5.4)	30.3 (5.8)	0.18
Body Mass Index (kg.m^−2^)	27.3 (22.0–31.5)	25.5 (21.0 –30.1)	0.38 *
Pre-eclampsia	14 (34.1%)	29 (36.3%)	0.84
Cesarian delivery	13 (31.7%)	28 (35.0%)	0.84
Smoking	4 (9.8%)	6 (7.5%)	0.73
Nulliparous	20 (48.8%)	40 (50.0%)	0.99
Family history of allergy	34 (82.9%)	54 (67.5%)	0.09
**Child**
Age (yr)	2.6 (1.7–3.8)	2.4 (1.5–3.8)	0.67 *
Male baby	21 (51.2%)	42 (52.5%)	0.99
Gestation at delivery (wk)	38.8 (1.8)	38.9 (1.3)	0.95 *
Birth weight (g)	3298 (491)	3365 (577)	0.86 *
Length at birth (cm)	49.8 (4.1)	50.7 (2.9)	0.32 *
Head circumference at birth (cm)	34.7 (3.8)	34.6 (1.9)	0.49 *
1 allergic disease	30 (73.2%)		
≥2 allergic diseases	11 (26.8%)		
Asthma	24 (58.5%)		
Atopic dermatitis	18 (43.9%)		
Food allergy	11 (26.8%)		
**Fetal size at mid-gestation**
Gestation at ultrasound (wk)	19.2 (0.7)	19.3 (0.8)	0.28
Head circumference (mm)	160.6 (8.9)	164.6 (10.4)	0.038
Biparietal diameter (mm)	43.3 (2.9)	44.1 (3.1)	0.16
Abdominal circumference (mm)	141.7 (9.0)	143.7 (12.1)	0.35
Femur length (mm)	29.8 (2.3)	30.0 (2.8)	0.69

*Data expressed as *N* (%), mean (SD), or median (Q1–Q3)*.

*Indicates that a Mann–Whitney *U*-test was performed.

**Table 2:** **Univariate logistic regression models for an outcome of an allergy vs. no allergy**.

Variable	Odds ratio (95% CI)	*P* value
Head circumference at mid-gestation per 1-mm increase	0.96 (0.92, 0.997)	0.037
Pre-eclampsia (N vs. Y)	0.85 (0.50, 1.47)	0.56
Nulliparity (N vs. Y)	1.14 (0.67, 1.95)	0.64
Gender (F vs. M)	0.99 (0.58, 1.69)	0.96
Cesarian delivery (N vs. Y)	1.28 (0.74, 2.21)	0.38
Smoking (N vs. Y)	1.16 (0.45, 2.99)	0.76
Maternal age per year increase	0.98 (0.93, 1.02)	0.34
Maternal BMI per unit increase	0.99 (0.95, 1.03)	0.58

Maternal and fetal characteristics were comparable in the mid-gestation analysis, excepting head circumference, which was significantly smaller in fetuses later affected by allergy [160.6 mm (SD = 8.9) vs. 164.6 mm (SD = 10.4), *p* = 0.038]. At birth, however, there was no difference in head circumference measures (allergy, median = 345 mm; no allergy, median = 345 mm, *p* = 0.73).

Univariate modeling revealed no significant association between pre-eclampsia and child allergy status (Table 2). Similarly, nulliparity, gender, cesarian delivery, smoking, maternal age, and BMI were not statistically associated with allergy status. Reported family history of allergy was comparable between families with allergic children, vs. non-allergic children (82.9 vs. 67.5%, *p* = 0.09). At mid-gestation, head circumference was the only anthropometric measure linked to allergy status in univariate modeling [OR = 0.96 (95% CI: 0.92–0.997), *p* = 0.037], which included biparietal diameter, femur length, and abdominal circumference measures.

**Figure 2:**
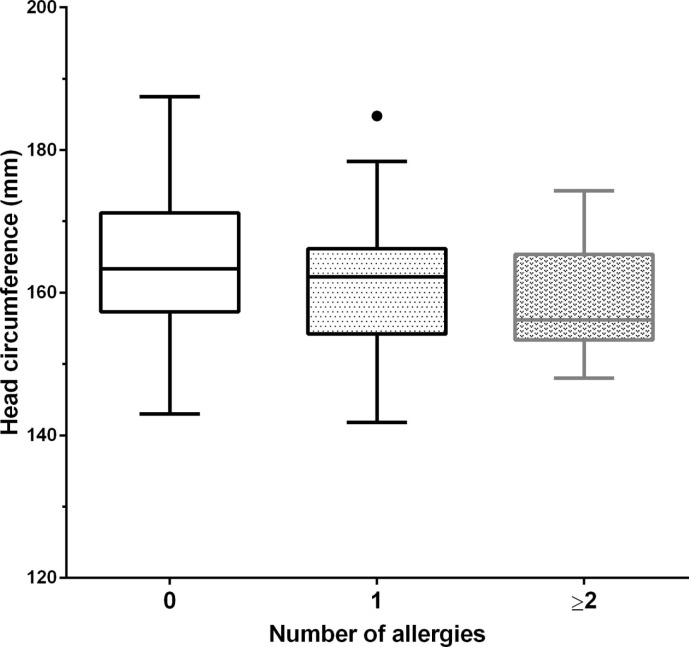
**Head circumference at mid-gestation vs. number of childhood allergies**. Tukey boxplot, whereby “∙” represents a data point greater than the 75th percentile plus 1.5 times the interquartile range. 0 allergies (*N* = 80), 1 allergy (*N* = 30), ≥2 allergies (*N* = 11). An inverse relationship was observed between mid-gestation head circumference and the number of childhood allergies (*p* = 0.033).

Multiple logistic regression revealed small fetal head circumference at mid-gestation to be significantly linked to childhood allergy, adjusted for gestation at ultrasound, with each decrease of 1 mm associated with an increased risk of 7% increase in risk of allergy (95% CI: 1–14%, *p* = 0.036). Furthermore, ordinal logistic regression analysis found fetal head circumference at mid-gestation to be related to the number of childhood allergies. For a patient with no allergies, each 1-mm increase in head circumference corresponds to a 7% increased risk of having an allergy. Similarly, for a patient with one allergy, each 1-mm increase in head circumference corresponds to a 7% increased risk of having two or more allergies. [Figure 2; OR = 0.93 (95% CI: 0.80–0.99), *p* = 0.033].

Head circumference at birth was not associated with allergic disease in univariate modeling [OR = 0.87 (95% CI: 0.73–1.04), *p* = 0.13]. Furthermore, head circumference at birth was not linked to allergy status in multivariate modeling, adjusted for gestation at delivery and birth weight [OR = 0.91 (95% CI: 0.72–1.15), *p* = 0.42]. In this multivariate model, childhood allergy status was also independent of gestation at delivery [OR = 0.87 (95% CI: 0.72–1.06), *p* = 0.16] and birth weight [OR = 1.00 (95% CI: 0.99–1.00), *p* = 0.97].

## Discussion

This is the first study to associate childhood allergy with decreased head circumference in fetal life. At mid-gestation, fetal head circumference was inversely related to the number of allergies in a given child, suggesting a dose–response in terms of degree of atopic predisposition and head growth *in utero*. In multiple logistic regression analyses, differences in head circumference remained significant after adjusting for gestational age.

Children in the allergy and no allergy groups were comparable for all maternal variables, including pre-eclamptic cases. Child variables were also comparable between groups, excepting head circumference at mid-gestation. Importantly, the median age of allergic children was relatively young in this study (2.6 years). Included children thus represent early onset allergy, and comparison with other studies should bear this consideration.

Changes in anthropometry at birth have been associated with several adult-onset disease states including hypertension (19) and coronary artery disease (20). Similarly, a growing body of evidence supports fetal programing in allergic disease. However, differences in methodology and contradictory findings, combined with restricted access to the fetus, have hindered efforts to delineate a clear mechanism. For instance, Fergusson et al., 1997 found increased head circumference at birth to be associated with asthma, but the same association was not observed for eczema or allergic rhinitis (3). On the other hand, Carrington et al., 2006 associated a small head circumference 10–15 days after birth with childhood wheeze (21). Such inconsistencies highlight a need for further research in this area. Nevertheless, fetal programing in atopic disease remains well supported, with increased head circumference at birth consistently linked to elevated IgE in childhood and adult life (1, 4, 22).

Decreased fetal head growth early *in utero* has been linked to asthma previously (23). However, that particular study utilized biparietal diameter measurements, which were not significantly different in our study. A separate cohort study found no association between fetal head circumference and atopic wheeze; however, early decreased head growth was linked to non-atopic wheeze at 3 years, perhaps the result of smaller airways (24). To our knowledge, no other studies have looked at early fetal growth and atopic disease, and further research is needed to clarify inconsistent findings. In view of the relatively young age of our cohort, a follow-up study is also warranted.

Previous contributors have proposed the “brain sparing” mechanism to link changes in fetal anthropometry with atopic disease. In line with this theory, the fetus may preferentially sustain brain growth at the expense of “less important” organs, including the thymus. Accordingly, increased head circumference has been considered an indirect marker of thymus undernutrition. However, in this study, childhood allergy was not associated with a statistical difference in neonatal head circumference. Combined with earlier and conflicting reports, a link between “brain sparing” and allergic disease is not well supported, and alternative mechanisms should be explored.

Aside from “brain sparing,” changes in neurotrophin exposure may link altered fetal growth to atopic disease. BDNF and other neurotrophins are important mediators of neuronal growth, maintenance, and differentiation (25). Alterations in neurotrophins, as seen in various neuropsychiatric disorders, are also linked to changes in brain growth and volume (26, 27). Beyond their primary functions in neurodevelopment, neurotrophins are upregulated in allergic inflammation (8). As well as promoting survival of eosinophils (28) and plasma cells (9), neurotrophins mediate chronic airway remodeling in asthma (29).

With a link between neurotrophins and atopic disease established, could altered fetal exposure to neurotrophins explain our findings? In humans, cord blood BDNF normally increases from mid-gestation onward (30). Accordingly, fetal neurodevelopment may greatly depend on neurotrophin levels in late gestation. Our finding of a small fetal head at mid-gestation, and a “normal”-sized head at birth, might thus reflect a compensatory increased effect of neurotrophins during this period. However, little is known about the biology of neurotrophins *in utero*, and studies are needed to further explore this hypothesis.

There were four reasons why pre-eclamptic pregnancies were initially selected and matched in this study. First, pre-eclampsia is associated with fetal growth restriction (31). Second, we have earlier shown fetal thymus size to be smaller in pre-eclampsia (32), with possible implications for child immune function. Third, limited evidence has linked pre-eclampsia with atopic sensitization (33). Finally, we have recently found an altered pattern of fetal head growth in pre-eclampsia, independent of growth restriction (Eviston et al., unpublished). However, despite these known associations, this study found no significant association between pre-eclampsia and childhood allergy.

This study is limited by its questionnaire-based design and the IgE-mediated food allergy component of the CECAQ is yet to be validated. A completed questionnaire response rate of 30.8% also raises the possibility of selection bias; however, only 11 persons actively declined participation following an attempt at telephone correspondence. Hence, the vast majority of incomplete questionnaires were due to an inability to contact mothers; an anticipated problem, since contact details were several years old in most instances. Mechanistically, our findings are plausible, but have not been confirmed by fetal biologic data. It is difficult to envisage a study design which would allow fetal blood sampling, given ethical and logistical concerns. Lastly, since the study design involved matching pre-eclamptic pregnancies, a potential for confounding bias was introduced; however, both the allergy and no allergy groups of children contained a comparable number of pre-eclamptic pregnancies and variables were similar between pre-eclamptic and non-pre-eclamptic pregnancies. Moreover, pre-eclampsia was unrelated to atopy in univariate analysis.

In conclusion, although these findings warrant replication, a small fetal head circumference at mid-gestation suggests that early fetal programing and exposures are important in the subsequent development of allergic disease.

## Author Contributions

AM: Study design, data collection, revision of manuscript, and final approval. DE: Manuscript preparation, statistics, and final approval. KM: Statistics, revision of manuscript, and final approval. DC and RN: Study design, revision of manuscript, and final approval.

## Conflict of Interest Statement

The authors declare that the research was conducted in the absence of any commercial or financial relationships that could be construed as a potential conflict of interest.
